# Prevalence of Depression and Anxiety Among Post-bariatric Surgery Patients: A Cross-Sectional Study

**DOI:** 10.7759/cureus.72399

**Published:** 2024-10-25

**Authors:** Abdullah N Alqifari, Sultan Alsaigh, Ghada Al Harbi, Jana Alnassar, Wamidh Alkhalifah, Raghad Alwehaibi, Khaled Alrakbi, Abdulmajeed Alkhamees, Hana N Alqifari

**Affiliations:** 1 Psychiatry, Qassim University, Buraydah, SAU; 2 Medicine and Surgery, King Fahad Specialist Hospital, Buraydah, SAU; 3 Psychiatry and Behavioral Sciences, Qassim University, Buraydah, SAU; 4 Medicine, Qassim University, Buraydah, SAU; 5 General Surgery, King Fahad Specialist Hospital, Buraydah, SAU; 6 Medicine, Medical Sciences, Qassim University, Buraydah, SAU; 7 Statistics and Operation Research, Medical Sciences, Qassim University, Buraydah, SAU

**Keywords:** anxiety, bariatric surgery, depression, obesity, saudi arabia

## Abstract

Introduction

Depression and anxiety are common mental health disorders that can significantly affect quality of life, particularly in patients following bariatric surgery. These psychological conditions are frequently observed after surgery and can have adverse consequences on recovery and health. This study aimed to assess the prevalence of anxiety and depression in post-bariatric surgery patients and to explore the relationship between bariatric surgery and the development of depressive and/or anxiety symptoms.

Methods

A cross-sectional study was conducted among patients who underwent bariatric surgery between June 1, 2019, and December 30, 2021, at King Fahad Specialist Hospital in Buraidah, Al Qassim, Saudi Arabia. Data collection occurred in two phases. Initially, medical records were reviewed to identify eligible patients, whose contact information was then entered into a digital database. Data collectors contacted patients via phone to obtain demographic details, chronic medical conditions, mental health history, BMI, surgical method, and symptoms of anxiety and depression. Anxiety and depression symptoms were assessed using the GAD-7 and PHQ-9 scales, respectively.

Results

The study included 182 post-bariatric surgery patients. The prevalence of depression was 3.8%. While 67.6% of participants reported no anxiety, a significant portion experienced anxiety at varying levels (20.9% mild, 6.6% moderate, and 4.9% severe). Depression was associated with younger age, single marital status, lower income, and smoking. Anxiety was linked to smoking, snoring, surgical complications, mental health history, recent stressors, and coexisting depression. Depression was more prevalent six months to two years post-surgery.

Conclusion

Psychological distress, particularly anxiety, is common among post-bariatric surgery patients. Routine psychiatric evaluation, both pre- and post-surgery, is recommended to prevent potential complications and improve patient outcomes.

## Introduction

Obesity has reached epidemic levels worldwide, presenting significant health challenges for both adults and children [1]. Compared to individuals of normal weight, those with obesity are more prone to developing a variety of mental health disorders, such as depressive disorder, and non-psychiatric conditions, including diabetes mellitus and cardiovascular disease [2]. Depression and anxiety are common among post-bariatric surgery patients, with nearly one in five patients experiencing depression postoperatively [3]. Depression levels tend to peak six to 12 months after surgery, though they may subside over time. Studies have reported varying rates of depression, ranging from 8.2% to 36% [4,5], while anxiety has been noted in approximately 11% of post-bariatric surgery patients [5]. Significant improvements in anxiety and depression symptoms have been observed as early as six weeks through three to six months postsurgery [6].

A systematic review and meta-analysis of 33 studies involving 101,223 patients across different countries reported a 15% prevalence of depression after bariatric surgery. Among those affected, 1.9%, 5.1%, and 64.9% experienced severe, moderate, and minimal depression, respectively [7]. Depression can negatively affect post-bariatric surgery outcomes, as it has been associated with reduced weight loss and an increased incidence of eating disorders. However, another systematic review of psychological outcomes from bariatric surgery indicated that, while many patients experience temporary relief from anxiety and depression symptoms, these may gradually return to pre-surgical levels over time [8].

In Saudi Arabia, several studies have also reported a significant prevalence of depression and anxiety among post-bariatric surgery patients. A cross-sectional study conducted at King Khalid University Hospital in Riyadh found that the rate of anxiety disorders ranged from 20.7% to 8.7%, while depressive disorders were reported between 46.9% and 4.4% [9]. Interestingly, the study found no significant change in anxiety and depressive symptoms over the short-, medium-, or long-term following surgery, though nearly 97% of patients expressed satisfaction with the surgery's outcomes [9].

In contrast, other studies have shown that symptoms of depression and anxiety can improve after bariatric surgery. A study by Aylward et al. [6] demonstrated significant improvement in anxiety and depression symptoms at six weeks and three to six months postsurgery. Similarly, a systematic review found that depressive and anxiety symptoms decreased for most patients within the first three years following surgery [10]. These findings suggest that bariatric surgery can have positive effects on mental health outcomes in the medium to long term.

Elevated depression levels following surgery have been linked to suboptimal outcomes, including insufficient weight loss or weight regain, comorbid psychopathology, reduced quality of life, and an increased risk of suicidal ideation and suicide [9]. Addressing postoperative depression is essential to ensuring the overall well-being and success of bariatric surgery patients. Furthermore, psychosocial factors have been shown to influence weight loss outcomes after surgery, with studies reporting improvements in depressive symptoms and quality of life, although social anxiety scores showed less improvement [11]. This highlights the complex relationship between mental health and weight loss outcomes in post-bariatric surgery patients.

To our knowledge, no previous studies have investigated the prevalence of depression in post-bariatric surgery patients in the Qassim region. Therefore, this study aims to determine the prevalence of depression and anxiety and their associated factors in post-bariatric surgery patients at King Fahad Specialist Hospital.

## Materials and methods

A cross-sectional study was conducted on patients who underwent bariatric surgery at King Fahad Specialist Hospital in Buraydah City, Al-Qassim region, Saudi Arabia, between June 1, 2019, and December 30, 2021. The study included all adult patients (18 years of age and older) who had undergone bariatric surgery during the specified period. Informed consent was obtained from all participants. Patients under the age of 18 or those who did not provide consent were excluded from the study.

A total of 182 patients were included and contacted by phone. Anxiety symptoms were assessed using the Generalized Anxiety Disorder-7 (GAD-7) scale, a validated tool for evaluating anxiety in the general population [12]. The GAD-7 is a seven-item questionnaire in which a score of 5 or less indicates mild anxiety, a score between 5 and 10 indicates moderate anxiety, and a score of 15 or above indicates severe anxiety. Depressive symptoms were evaluated using the Patient Health Questionnaire-9 (PHQ-9). The PHQ-9 consists of nine items, each scored from 0 to 3, with thresholds for mild, moderate, and severe depression set at 5, 15, and 20, respectively [13].

An electronic self-administered questionnaire was developed in two parts, based on a review of relevant literature. The first part collected demographic information, including age, nationality, gender, marital status, education, household income, and place of residence. Additionally, socioeconomic factors - such as major life events negatively affecting psychological health (e.g., job loss, financial difficulties, divorce, retirement, or grief) - were assessed through both structured questions and open-ended responses. Chronic medical conditions such as diabetes, hypertension, dyslipidemia, family history of psychiatric illness, body mass index (BMI), and type of bariatric surgery were also evaluated. The second part of the questionnaire included the GAD-7 and PHQ-9 scales to assess patients' anxiety and depression levels.

Statistical analysis

Data analysis was performed using Statistical Product and Service Solutions (SPSS, version 26.0; IBM SPSS Statistics for Windows, Armonk, NY). Descriptive statistics, including means, standard deviations, and frequencies, were calculated to summarize the characteristics of the study population. Chi-square tests were used to assess the statistical significance of associations between sociodemographic factors and mental health outcomes, such as depression and anxiety. A p-value of less than 0.05 was considered statistically significant. Additionally, logistic regression analysis was conducted to examine the impact of various factors on the likelihood of experiencing depression and anxiety. All analyses were conducted using two-tailed tests.

Ethical considerations

Ethical approval for this study was obtained from the local committee for research ethics at Qassim. Participants were informed about the purpose of the research, and electronic consent was obtained prior to completing the questionnaire. Data were initially identified and then coded in an Excel database using unique identification numbers to ensure confidentiality. The data were securely stored on a password-protected laptop, accessible only to the research team for analysis. No identifying information was used in the publications, with only summary statistics being presented. Participants were informed of the study's purpose, the potential risks and benefits of participation, and their right to withdraw from the study at any time without penalty. Informed consent was obtained from all participants before their involvement in the study.

## Results

Participant demographics

The study sample consisted of 182 participants, including 104 women (57.1%) and 78 men (42.9%), with the vast majority (99.5%, n=181) being Saudi nationals. Participants were distributed across various age groups, with the largest proportion (33.5%, n=61) falling between 30 and 39 years of age. Regarding marital status, 70.3% (n=128) were married, 23.6% (n=43) were single, and 6.0% (n=11) were divorced. Educational attainment varied, with the largest group (47.8%, n=87) having a high school education or lower, followed by 36.3% (n=66) with a bachelor's degree. A smaller proportion held diplomas (11.5%, n=21) or pursued higher studies (4.4%, n=8). The socio-demographic characteristics of the study population are summarized in Table 1.

**Table 1:** Socio-demographic characteristics of bariatric surgery patients (N=182)

Socio-demographic characteristics		N (%)
Category	Variable	
Gender	Male	78 (42.9%)
	Female	104 (57.1%)
Nationality	Saudi	181 (99.5%)
	Non-Saudi	1 (0.5%)
Age Group	Less than 30 years old	46 (25.3%)
	30-39 years old	61 (33.5%)
	40-49 years old	43 (23.6%)
	50 years old or above	32 (17.6%)
Marital Status	Single	43 (23.6%)
	Married	128 (70.3%)
	Divorced	11 (6.0%)
Educational Level	High school or lower	87 (47.8%)
	Diploma	21 (11.5%)
	Bachelor's degree	66 (36.3%)
	Higher studies	8 (4.4%)
Employment Status/Sector	Unemployed	82 (45.1%)
	Private sector	43 (23.6%)
	Government sector	57 (31.3%)
Approximate Monthly Household Income in Saudi Riyals	Less than 5000 SAR	64 (35.2%)
	5000-10,000 SAR	70 (38.5%)
	10,000-20,000 SAR	35 (19.2%)
	Above 20,000 SAR	13 (7.1%)
Are you a smoker?	Yes	25 (13.7%)
	No	151 (83.0%)
	Former smoker	6 (3.3%)
Have you been diagnosed with any medical condition?	Yes	65 (35.7%)
	No	117 (64.3%)
What type of surgical procedure did you undergo?	Gastric bypass surgery (gastric diversion)	8 (4.4)
	Gastric sleeve surgery (stomach resection)	174 (95.6)
When was the surgery conducted?	Within the past six months	3 (1.6%)
	6 months-1 year	7 (3.8%)
	1 year-2 year	42 (23.1%)
	2 year-3 year	31 (17.0%)
	3 year-4 year	39 (21.4%)
	More than 4 years ago	60 (33.0%)
Did you encounter any problems or complications related to the surgery	Yes	30 (16.5%)
	No	152 (83.5%)
How satisfied are you with the results of the surgery	Neutral	7 (3.8%)
	Not satisfied	3 (1.6%)
	Satisfied to some extent	37 (20.3%)
	Very satisfied	135 (74.2%)
Did you consult a psychiatrist before the surgery?	No	174 (95.6%)
	Yes	8 (4.4%)
Have you been diagnosed with any mental illness?	No	175 (96.2%)
	Yes	7 (3.8%)
Are there any family members who have been diagnosed with a mental or psychological illness?	No	166 (91.2%)
	Yes	16 (8.8%)
Have you ever used amphetamines and illegal drugs?	No	180 (98.9%)
	Yes	2 (1.1%)
Have you experienced any recent family, social, work-related, or economic problems?	No	143 (78.6%)
	Yes	39 (21.4%)

Employment status was diverse, with 45.1% (n=82) of participants being unemployed, 31.3% (n=57) employed in the government sector, and 23.6% (n=43) employed in the private sector. Monthly household income also varied, with the largest group (38.5%, n=70) earning between 5,000 and 10,000 SAR. A minority (13.7%, n=25) of participants were current smokers, while the majority (83.0%, n=151) reported no history of smoking. Over one-third of participants (35.7%, n=65) had pre-existing medical conditions.

The vast majority of patients (95.6%, n=174) underwent gastric sleeve surgery, with only a small number (4.4%, n=8) receiving gastric bypass surgery. Most surgeries were performed more than four years prior to the study (33.0%, n=60). Postsurgical complications were reported by 16.5% (n=30) of participants.

Satisfaction with surgical outcomes was high, with 74.2% (n=135) reporting they were very satisfied, and 20.3% (n=37) somewhat satisfied. Only 3.8% (n=7) were neutral or dissatisfied. Additionally, the majority of participants (95.6%, n=174) did not consult a psychiatrist before surgery. While most participants (96.2%, n=175) had no diagnosis of mental illness, a small percentage (3.8%, n=7) reported having a mental health diagnosis. Additionally, 8.8% (n=16) reported a family history of mental illness. The use of amphetamines and illegal drugs was rare among the participants (1.1%, n=2). However, a significant proportion (21.4%, n=39) reported experiencing recent family, social, work-related, or economic problems.

Prevalence of depression and anxiety

In terms of mental health outcomes, the prevalence of depression was relatively low, with only 3.8% of participants meeting the diagnostic criteria for depression (at least five symptoms, including depressed mood or loss of interest) (see Table 2).

**Table 2:** Prevalence of depression and levels of anxiety among post-bariatric surgery patients

Disease	Categorical	N (%)
Depression (at least 5 symptoms of depression with one of them: decreased interest or depressed mood)	No	175 (96.2%)
	Yes	7 (3.8%)
Levels of anxiety	None	123 (67.6%)
	Mild	38 (20.9%)
	Moderate	12 (6.6%)
	Severe	9 (4.9%)

In contrast, anxiety levels displayed greater variability. While 67.6% of participants reported no anxiety, 20.9% experienced mild anxiety, 6.6% had moderate anxiety, and 4.9% reported severe anxiety. These findings indicate that, although depression is uncommon in this population, anxiety is more prevalent and varies in severity. The analysis of sociodemographic characteristics and their associations with anxiety levels revealed several significant relationships. Higher anxiety levels were significantly associated with smoking status (p=0.005), preoperative snoring related to obesity (p=0.026), surgical complications (p=0.031), mental illness diagnosis (p=0.041), family history of mental illness (p<0.001), recent stressors (p<0.001), and the presence of depression (p<0.001) (Table 3).

**Table 3:** Association between socio-demographic characteristics of the study participants and levels of anxiety using the likelihood ratio test **significant at p<0.05 level. $p-value was calculated using the likelihood ratio test

		Levels of Anxiety				p-value^$^
		N (%)				
Category	Variable	None	Mild	Moderate	Severe	
Gender	Male	52 (42.3%)	15 (39.5%)	4 (33.3%)	7 (77.8%)	0.163
	Female	71 (57.7%)	23 (60.5%)	8 (66.7%)	2 (22.2%)	
Nationality	Saudi	122 (99.2%)	38 (100%)	12 (100%)	9 (100%)	0.923
	Non-Saudi	1 (0.8%)	0 (0.0%)	0 (0.0%)	0 (0.0%)	
Age Group	Less than 30 years old	27 (22.0%)	11 (28.9%)	4 (33.3%)	4 (44.4%)	0.382
	30-39 years old	44 (35.8%)	9 (23.7%)	5 (41.7%)	3 (33.3%)	
	40-49 years old	28 (22.8%)	12 (31.6%)	3 (25.0%)	0 (0.0%)	
	50 years old or above	24 (19.5%)	6 (15.8%)	0 (0.0%)	2 (22.2%)	
Marital Status	Single	26 (21.1%)	10 (26.3%)	5 (41.7%)	2 (22.2%)	0.279
	Married	90 (73.2%)	26(68.4%)	7 (58.3%)	5 (55.6%)	
	Divorced	7 (5.7%)	2 (5.3%)	0 (0.0%)	2 (22.2%)	
Educational Level	High school or lower	58 (47.2%)	19 (50.0%)	7 (58.3%)	3 (33.3%)	0.883
	Diploma	15 (12.2%)	5 (13.2%)	0 (0.0%)	1 (11.1%)	
	Bachelor's degree	44 (35.8%)	13 (34.2%)	4 (33.3%)	5 (55.6%)	
	Higher studies	6 (4.9%)	1 (2.6%)	1 (8.3%)	0 (0.0%)	
Employment Status/Sector	Unemployed	54 (43.9%)	19 (50.0%)	6 (50.0%)	3 (33.3%)	0.138
	Private sector	23 (18.7%)	13 (34.2%)	4 (33.3%)	3 (33.3%)	
	Government sector	46 (37.4%)	6 (15.8%)	2 (16.7%)	3 (33.3%)	
Approximate Monthly Household Income in Saudi Riyals	Less than 5000 SAR	37 (30.1%)	18 (47.4%)	5 (41.7%)	4 (44.4%)	0.418
	5000-10,000 SAR	53 (43.1%)	11 (28.9%)	4 (33.3%)	2 (22.2%)	
	10,000-20,000 SAR	24 (19.5%)	7 (18.4%)	1 (8.3%)	3 (33.3%)	
	Above 20,000 SAR	9 (7.3%)	2 (5.3%)	2 (16.7%)	0 (0.0%)	
Are You a Smoker?	Yes	11 (8.9%)	7 (18.4%)	1 (8.3%)	6 (66.7%)	0.005**
	No	109 (88.6%)	29 (76.3%)	10 (83.3%)	3 (33.3%)	
	Former smoker	3 (2.4%)	2 (5.3%)	1 (8.3%)	0 (0.0%)	
Have You Been Diagnosed with Any Medical Condition?	Yes	42 (34.1%)	14 (36.8%)	5 (41.7%)	4 (44.4%)	0.888
	No	81 (65.9%)	24 (63.2%)	7 (58.3%)	5 (55.6%)	
Snoring Before the Surgery due to Obesity	1	36 (29.3)	17 (44.7)	7 (58.3)	3 (33.3)	0.026**
	2	6 (4.9)	0 (0.0)	1 (8.3)	3 (33.3)	
	3	2 (1.6)	0 (0.0)	0 (0.0)	0 (0.0)	
	4	79 (64.2)	21 (55.3)	4 (33.3)	3 (33.3)	
What Type of Surgical Procedure Did You Undergo?	Gastric bypass surgery(gastric diversion)	4 (3.3%)	2 (5.3%)	2 (16.7%)	0 (0.0%)	0.16
	Gastric sleeve surgery (stomach resection)	119 (96.7%)	36 (94.7%)	10 (83.3%)	9 (100%)	
When Was the Surgery Conducted?	Within the past six months	3 (2.4%)	0 (0.0%)	0 (0.0%)	0 (0.0%)	0.971
	6 months-1 year	4 (3.3%)	2 (5.3%)	1 (8.3%)	0 (0.0%)	
	1 year-2 year	26 (21.1%)	11 (28.9%)	2 (16.7%)	3 (33.3%)	
	2 year-3 year	23 (18.7%)	5 (13.2%)	2 (16.7%)	1 (11.1%)	
	3 year-4 year	24 (19.5%)	9 (23.7%)	3 (25.0%)	3 (33.3%)	
	More than 4 years ago	43 (35.0%)	11 (28.9%)	4 (33.3%)	2 (22.2%)	
Did You Encounter any Problems or Complications Related to the Surgery?	Yes	18 (14.6%)	4 (10.5%)	4 (33.3%)	4 (44.4%)	0.031**
	No	105 (85.4%)	34 (89.5%)	8 (66.7%)	5 (55.6%)	
How Satisfied Are You with the Results of the Surgery?	Neutral	3 (2.4%)	2 (5.3%)	1 (8.3%)	1 (11.1%)	0.112
	Not satisfied	2 (1.6%)	1 (2.6%)	0 (0.0%)	0 (0.0%)	
	Satisfied to some extent	21 (17.1%)	10 (26.3%)	6 (50.0%)	0 (0.0%)	
	Very satisfied	97 (78.9%)	25 (65.8%)	5 (41.7%)	8 (88.9%)	
Did You Consult a Psychiatrist Before the Surgery?	No	116 (94.3%)	37 (97.4%)	12 (100%)	9 (100%)	0.628
	Yes	7 (5.7%)	1 (2.6%)	0 (0.0%)	0 (0.0%)	
Have You Been Diagnosed with any Mental Illness?	No	119 (96.7%)	38 (100%)	10 (83.3%)	8 (88.9%)	0.041**
	Yes	4 (3.3%)	0 (0.0%)	2 (16.7%)	1 (11.1%)	
Are There Any Family Members Who Have Been Diagnosed with a Mental or Psychological Illness?	No	114 (92.7%)	37 (97.4%)	7 (58.3%)	8 (88.9%)	<0.05**
	Yes	9 (7.3%)	1 (2.6%)	5 (41.7%)	1 (11.1%)	
Have You Ever Used Amphetamines and Illegal Drugs?	No	121 (98.4%)	38 (100%)	12 (100%)	9 (100%)	0.809
	Yes	2 (1.6%)	0 (0.0%)	0 (0.0%)	0 (0.0%)	
Have You Experienced Any Recent Family, Social, Work-Related, or economic Problems?	No	108 (87.8%)	28 (73.7%)	4 (33.3%)	3 (33.3%)	<0.05**
	Yes	15 (12.2%)	10 (26.3%)	8 (66.7%)	6 (66.7%)	
Levels of Depression	None	94 (76.4%)	11 (28.9)	1 (8.3)	0 (0.0)	<0.05**
	Mild	24 (19.5%)	14 (36.8%)	6 (50.0%)	3 (33.3%)	
	Moderate	5 (4.1%)	9 (23.7%)	2 (16.7%)	2 (22.2%)	
	Moderate to severe	0 (0.0%)	4 (10.5%)	3 (25.0%)	4 (44.4%)	

In contrast, several sociodemographic factors were not significantly associated with anxiety levels, including gender (p=0.163), nationality (p=0.923), age group (p=0.382), marital status (p=0.279), educational level (p=0.883), employment sector (p=0.138), approximate monthly household income (p=0.418), diagnosis of a medical condition (p=0.888), type of surgical procedure (p=0.160), timing of surgery (p=0.971), satisfaction with surgical outcomes (p=0.112), pre-surgery psychiatric consultation (p=0.628), and amphetamine or drug use (p=0.809).

The likelihood ratio test revealed significant associations between several sociodemographic factors and depression. Younger age (<30 years, p=0.046), single marital status (p=0.020), lower income (less than 5,000 SAR, p=0.020), and current smoking status (p=0.047) were all significantly associated with an increased likelihood of depression. No significant associations were found for gender, nationality, education level, or employment sector (p>0.05). Additionally, the time since surgery was found to be a significant factor in the development of depression (p<0.05). Participants who had undergone surgery between six months and two years prior exhibited a higher prevalence of depression compared to those who had surgery at other timeframes (Table 4).

**Table 4:** Association between variables and diagnosis of Depression using likelihood ratio test **significant at p<0.05 level. $p-value was calculated using the Likelihood ratio test

Category	Variable	No Depression, n (%)	Depression, n (%)	p-value^$^
Gender	Male	74 (42.3%)	4 (57.1%)	0.697
	Female	101 (57.7%)	3 (42.9%)	
Nationality	Saudi	174 (99.4%)	7 (100%)	0.779
	Non-Saudi	1 (0.6%)	0 (0.0%)	
Age Group	Less than 30 years old	41 (23.4%)	5 (71.4%)	0.046**
	30-39 years old	60 (34.3%)	1 (14.3%)	
	40-49 years old	42 (24.0%)	1 (14.3%)	
	50 years old or above	32 (18.3%)	0 (0.0%)	
Marital Status	Single	38 (21.7%)	5 (71.4%)	0.020**
	Married	126 (72.0%)	2 (28.6%)	
	Divorced	11 (6.3%)	0 (0.0%)	
Educational Level	High school or lower	85 (48.6%)	2 (28.6%)	0.444
	Diploma	19 (10.9%)	2 (28.6%)	
	Bachelor's degree	63 (36.0%)	3 (42.9%)	
	Higher studies	8 (4.6%)	0 (0.0%)	
Employment Status/Sector	Unemployed	70 (45.1%)	3 (42.9%)	0.412
	Private sector	40 (22.9%)	3 (42.9%)	
	Government sector	56 (32.0%)	1 (14.3%)	
Approximate Monthly Household Income in Saudi Riyals	Less than 5000 SAR	58 (33.1%)	6 (85.7%)	0.020**
	5000 - 10,000 SAR	69 (39.4%)	1 (14.3%)	
	10,000 - 20,000 SAR	35 (20.0%)	0 (0.0%)	
	Above 20,000 SAR	13 (7.4%)	0 (0.0%)	
Are you a smoker?	Yes	22 (12.6%)	3 (42.9%)	0.047**
	No	148 (84.6%)	3 (42.9%)	
	Former smoker	5 (2.9%)	1 (14.3%)	
Have you been diagnosed with any medical condition?	Yes	63 (36.0%)	2 (28.6%)	0.683
	No	112 (64.0%)	5 (71.4%)	
What type of surgical procedure did you undergo?	Gastric bypass surgery (gastric diversion)	7 (4.0%)	1 (14.3%)	0.718
	Gastric sleeve surgery (stomach resection)	168 (96.0%)	6 (85.7%)	
When was the surgery conducted?	Within the past six months	3 (1.7%)	0 (0.0%)	0.001**
	6 months - 1 year	5 (2.9%)	2 (28.6%)	
	1 year- 2year	37 (21.1%)	5 (71.4%)	
	2 year- 3year	31 (17.7%)	0 (0.0%)	
	3 year-4year	39 (2.3%)	0 (0.0%)	
	More than 4 years ago	60 (34.3%)	0 (0.0%)	
Did you encounter any problems or complications related to the surgery?	Yes	27 (15.4%)	3 (42.9%)	0.092
	No	148 (84.6%)	4 (57.1%)	
How satisfied are you with the results of the surgery?	Neutral	7 (4.0)	0 (0.0%)	0.478
	Not satisfied	3 (1.7%)	0 (0.0%)	
	Satisfied to some extent	34 (19.4%)	3 (42.9%)	
	Very satisfied	131 (74.9%)	4 (57.1%)	
Did you consult a psychiatrist before the surgery?	No	167 (95.4%)	7 (100%)	0.423
	Yes	8 (4.6%)	0 (0.0%)	
Have you been diagnosed with any mental illness?	No	168 (96.0%)	7 (100%)	0.454
	Yes	7 (4.0%)	0 (0.0%)	
Are there any family members who have been diagnosed with a mental or psychological illness?	No	161 (92.0%)	5 (71.4%)	0.121
	Yes	14 (8.0%)	2 (28.6%)	
Have you ever used amphetamines and illegal drugs?	No	173 (98.9%)	7 (100%)	0.691
	Yes	2 (1.1%)	0 (0.0%)	
Have you experienced any recent family, social, work-related, or economic problems?	No	138 (78.9%)	5 (71.4%)	0.65
	Yes	37 (21.1%)	2 (28.6%)	

A strong association was also observed between the presence of depression and self-reported impairment in daily functioning (p<0.001) (Table 5).

**Table 5:** Difficulty in performing daily responsibilities based on depression status **significant at p<0.05 level

	How difficult have these problems made it for you to perform your work, study or take care of your household responsibilities or get along with people; N (%)				Likelihood ratio test
	Not difficult at all	Somewhat difficult	Very difficult	Extremely difficult	p-value
No Depression	150 (100)	20 (76.9)	3 (75.0)	2 (100)	<0.05^**^
Depression	0 (0.0)	6 (23.1)	1 (25.0)	0 (0.0)	

All participants without depression reported no difficulty in daily activities, while those identified as depressed reported experiencing at least some level of impairment. This finding highlights the substantial impact of depression on individuals' ability to function effectively in their daily lives.

Binary logistic regression analysis

To further examine the relationship between sociodemographic characteristics and depression, binary logistic regression analysis was performed. While most sociodemographic factors were not significant predictors, monthly household income was significantly associated with depression (B=-2.327, Wald=4.240, df=1, p=0.039), indicating that lower household income is linked to higher odds of experiencing depression (Table 6).

**Table 6:** Binary logistic regression analysis

	B	S.E.	Wald	df	Sig.	Exp(B)
Gender	-0.181	1.122	0.026	1	0.872	0.834
Nationality	-13.959	40192.99	0	1	1	0
Age group	-0.887	0.741	1.431	1	0.232	0.412
Marital Status	-0.835	1.129	0.547	1	0.459	0.434
Educational Level	0.101	0.533	0.036	1	0.849	1.107
Employment Sector	0.393	0.687	0.327	1	0.567	1.481
Approximate Monthly Household Income in Saudi Riyals	-2.327	1.13	4.24	1	0.039	0.098
Smoking	-0.264	1.123	0.055	1	0.814	0.768
Constant	16.984	40192.99	0	1	1	23761755

## Discussion

To our knowledge, this is the first study to assess the rates of depression and anxiety among post-bariatric surgery patients in the Qassim region. In Saudi Arabia, anxiety disorders are the most prevalent mental health condition (12.3%), followed by mood disorders (6.8%) [14]. Contrary to initial expectations, we found a relatively low prevalence of depression in our study population, with only 3.8% of participants meeting the diagnostic criteria for depression. This contrasts with previous research, which reported significantly higher rates of depression in post-bariatric patients, with 29.4% experiencing mild depression, 11.2% moderate depression, 8.2% moderately severe depression, and 4.4% severe depression [9]. The discrepancy may be due to methodological differences between the studies, including variations in sample size, data collection methods, and diagnostic tools used.

In the Saudi context, cultural norms around body image and eating behaviors play a significant role in shaping mental health outcomes post-bariatric surgery. Research indicates that Saudi women exhibit higher rates of bulimic behaviors compared to anorexic behaviors, which may be linked to cultural acceptance of overeating​ [15]. This creates a unique environment where societal pressures to achieve an idealized body image can lead to psychological distress. These expectations, when unmet, can exacerbate feelings of anxiety and depression, particularly in post-surgical patients. Therefore, it is crucial to explore how these cultural factors impact mental health recovery and to develop culturally sensitive psychological support for bariatric patients.

In contrast, anxiety levels in our study showed greater variability. While the majority of participants (67.6%) reported no anxiety, 20.9% experienced mild anxiety, 6.6% moderate anxiety, and 4.9% severe anxiety. The rates of mild anxiety were consistent with previous findings (both around 20%), but our rates of moderate and severe anxiety were lower than those reported in earlier research, where 11.2% and 8.7% of participants, respectively, exhibited these symptoms [9]. This suggests that, while anxiety is a concern for some post-bariatric patients, the distribution of anxiety severity may vary across different studies and populations.

Previous studies have also shown higher rates of depression among females compared to males, particularly in obese populations [9,16]. However, our study did not identify a significant gender difference in depression levels among post-bariatric surgery patients. This discrepancy may be related to methodological differences, including our adherence to strict diagnostic criteria. Notably, a significant gender difference in depression emerged only when PHQ-9 symptoms were scored without requiring at least five core symptoms, such as decreased interest and depressed mood. This finding underscores the importance of using established diagnostic criteria, such as those of the PHQ-9, to ensure accurate assessment and comparison of depression rates across genders in this population.

Our findings align with existing literature, particularly regarding the significant association between depression and younger age. In our study, individuals under 30 years of age were significantly more likely to experience depression (p=0.046). This observation is supported by a cross-sectional study conducted at King Khalid University Hospital in Riyadh, which reported a negative correlation between increasing age and depression scores (PHQ-9). Specifically, individuals aged 30-39 were 18.3% less likely to experience depression compared to those under 30 years of age (p=0.021). This trend continued with individuals aged 40-49 showing a 23.8% reduced likelihood of depression (p=0.012), and those over 50 years demonstrating a 33% reduced likelihood (p<0.001) [9]. These converging findings emphasize the importance of considering age as a key factor when addressing depression in post-bariatric surgery patients. Marital status also demonstrated a significant association with depression in our study, with single individuals exhibiting higher depression levels than their married counterparts. This contrasts with previous research that did not identify a significant correlation between marital status and depression scores [9].

Although the majority of surgeries in our study were successful and free from complications, patients who experienced surgical complications reported significantly higher anxiety levels compared to those who did not. Interestingly, the type of bariatric surgery did not have a significant impact on anxiety levels, consistent with previous studies [9,17]. However, a study by Lu et al. using the National Health Insurance Research Database of Taiwan, which included over 2,300 patients, showed that the risk of major depression increased 2.36-fold with malabsorptive surgeries and 1.38-fold with restrictive surgeries [18]. Despite these risks, gastric resection has been shown to improve health-related quality of life (HRQL). A controlled clinical trial in Swedish Obese Subjects (SOS) intervention, which followed 487 surgical cases and their matched controls over two years, found that patients with poor HRQL prior to gastric restriction surgery experienced significant improvements, whereas those receiving conventional treatment showed only minor changes [19].

Our current research showed a significant association between smoking and higher levels of depression and anxiety. This finding is consistent with the SOS, which examined the long-term effects of bariatric surgery on health and quality of life [19]. Our research revealed a significant association between anxiety and preoperative snoring in obese individuals (p=0.026). This finding aligns with the study by Alshammari et al. that reported that post-bariatric surgery patients with a history of sleep apnea were significantly more prone to anxiety (19.1 times more) compared to those without sleep apnea, both before and after surgery (p=0.027) [9]. Our study found that 74.2% of bariatric surgery patients reported satisfaction with their outcomes. While lower than the rate in Riyadh (97%) [9], this result is higher than those found in similar studies in Taif (42.6%) [20] and Najran (66.9%) [21], and still suggests a positive impact of bariatric surgery on overall well-being.

One of the limitations of this study is the reliance on self-reported data for assessing anxiety and depression. This approach may introduce response bias, as participants may underreport symptoms due to stigma or overestimate their severity. Additionally, the cross-sectional design limits our ability to assess causality between bariatric surgery and the development of mental health disorders. Other limitations include the small sample size, which may have affected the generalizability of our findings, and the single-center design, which may limit the generalizability to other populations or settings. Given the higher prevalence of depression among younger, single patients, we recommend integrating targeted mental health interventions, such as cognitive-behavioral therapy (CBT) or stress management programs, for these groups pre- and post-operatively. These interventions could help mitigate the psychological challenges faced by this demographic, potentially improving both mental health outcomes and overall recovery. Future studies should explore the effectiveness of mental health interventions, such as psychotherapy and medication, in improving long-term postoperative outcomes. This could include randomized controlled trials investigating whether early intervention for anxiety and depression can reduce the risk of postoperative complications and improve overall quality of life.

## Conclusions

Anxiety and depression are common after bariatric surgery, with smoking significantly associated with both. Younger age and single status correlate with depression, while surgical complications and preoperative snoring are linked to anxiety. Patients with these risk factors should receive a psychiatric evaluation, and smokers should be offered cessation programs. In conclusion, addressing mental health concerns, particularly anxiety and depression, is crucial for optimizing postoperative outcomes in bariatric surgery patients. It is imperative that healthcare systems incorporate routine psychological evaluations as a standard part of pre- and post-surgical care to enhance both physical and psychological recovery.

## Appendices

The prevalence of anxiety and depression among patients following bariatric surgery at King Fahad Specialist Hospital in Buraydah is the focus of this study.

Greetings and peace be upon you; thank you for visiting the research page. Below are some important details regarding this study that should be understood and agreed upon in order to participate:

Research title

Prevalence of Anxiety and Depression Among Patients Following Bariatric Surgery at King Fahad Specialist Hospital in Buraidah.

Objective

This research aims to measure the prevalence of depression, anxiety, and factors associated with them among patients after bariatric surgery at King Fahad Specialist Hospital.

Risks

This study does not involve any risks that may have a negative impact on the individual participating in it.

Benefits

There are no personal benefits to the individual as a result of participating in this study.

Confidentiality

The researcher guarantees the confidentiality of the information obtained from the participating individual, and this information will only be used for research and statistical purposes. The individual has the right to decline participation in this study or withdraw from it after participating, without affecting the type or quality of healthcare and education services provided to them. Participating in this study will not involve any financial costs or compensation for the individual.

Your participation in this questionnaire indicates your agreement to participate in this study, knowing that you can withdraw while completing this questionnaire. To proceed, please select "Agree" for further follow-up.

Gender

Male

Female

Nationality

Saudi

Non-Saudi

Age group

Less than 30 years old

30-39 years old

40-49 years old

50 years old or above

Marital status

Single

Married

Divorced

Educational level

High school or lower diploma

Bachelor's degree

Higher studies

Employment sector

Unemployed

Private sector

Government sector

Approximate monthly household income in Saudi Riyals

Less than 5000 SAR

5000 - 10,000 SAR

10,000 - 20,000 SAR

Above 20,000 SAR

Are you a smoker?

Yes

No

Former smoker

Have you been diagnosed with any medical condition?

Yes

No

Select any applicable medical conditions:

Heart and cardiovascular diseases

Diabetes

Thyroid disorders

High blood pressure

Asthma and respiratory diseases

High cholesterol

Rheumatoid arthritis

Other (chronic kidney disease, arthritis, lupus, psoriasis, spinal disorders, skin rash, vitamin deficiency)

What health problems have you experienced due to obesity?

Sleep apnea:

Yes, but it improved and disappeared after surgery.

Yes, but there was no change compared to before surgery. Yes, it worsened and intensified after surgery.

No, I didn't experience it.

Snoring:

Yes, but it improved and disappeared after surgery.

Yes, but there was no change compared to before surgery. Yes, it worsened and intensified after surgery.

No, I didn't experience it.

Joint pain and problems:

Yes, but it improved and disappeared after surgery.

Yes, but there was no change compared to before surgery. Yes, it worsened and intensified after surgery.

No, I didn't experience it.

Ovarian cysts:

Yes, but it improved and disappeared after surgery.

Yes, but there was no change compared to before surgery. Yes, it worsened and intensified after surgery.

No, I didn't experience it.

Body mass index (BMI) before surgery:

30% - 35%

35% - 40%

Above 40%

Body mass index (BMI) after surgery:

Less than 20%

20% - 25%

25% - 30%

30% - 35%

35% - 40%

Above 40%

Did your body mass index (BMI) improve after surgery?

Yes

No

What type of surgical procedure did you undergo?

Gastric bypass surgery (gastric diversion)

Gastric sleeve surgery (stomach reduction)

When was the surgery conducted?

Within the past six months

6 months - 1 year

1 year - 2 years

2 years - 3 years

3 years - 4 years

More than 4 years ago

Did you encounter any problems or complications related to the surgery?

Yes

No

How satisfied are you with the results of the surgery?

Not satisfied at all

Not satisfied, Neutral

Satisfied to some extent Very satisfied

Did you consult a psychiatrist before the surgery?

No

Yes

When did you consult a psychiatrist?

I did, but I can't remember when

Yes, within less than a year Yes, prior to one to three years

Yes, prior to four years before the surgery

Have you been diagnosed with any mental illness?

No

Yes

What are the psychiatric diagnoses you have?

Obsessive-compulsive disorder

Schizophrenia

Panic attacks and social phobia

Personality disorders

Depression

Anxiety disorders

Other mental illnesses

Are there any family members who have been diagnosed with a mental or psychological illness?

No

Yes

What are their psychiatric diagnoses?

Schizophrenia

Panic attacks

Obsessive-compulsive disorder

Attention deficit hyperactivity disorder (ADHD)

Depression

Anxiety disorder

Have you ever used amphetamines and illegal drugs?

No

Yes

Have you experienced any recent family, social, work-related, or economic problems?

No

Yes

How frequently have you been bothered by any of the following problems in the past four weeks?

Feeling tense, nervous, or anxious

Not at all

Several days

More than half the days

Nearly every day

Not being able to stop or control worrying

Not at all

Several days

More than half the days

Nearly every day

Worrying too much about different things

Not at all

Several days

More than half the days

Nearly every day

Having trouble relaxing

Not at all

Several days

More than half the days

Nearly every day

Feeling restless to the point it's hard to sit still

Not at all

Several days

More than half the days

Nearly every day

Easily becoming annoyed or irritable

Not at all

Several days

More than half the days

Nearly every day

Feeling afraid as if something awful might happen to you

Not at all

Several days

More than half the days

Nearly every day

How difficult have these problems made it for you to perform your work, study, or take care of your household responsibilities, or get along with people?

Not difficult at all

Somewhat difficult

Very difficult

Extremely difficult

How frequently have you been bothered by any of the following problems in the past four weeks?

Little interest or pleasure in doing things?

Not at all

Several days

More than half the days

Nearly every day

Feeling down, depressed, or hopeless?

Not at all

Several days

More than half the days

Nearly every day

Trouble falling or staying asleep, or sleeping too much?

Not at all

Several days

More than half the days

Nearly every day

Feeling tired or having little energy?

Not at all

Several days

More than half the days

Nearly every day

Poor appetite or overeating?

Not at all

Several days

More than half the days

Nearly every day

Feeling bad about yourself - or that you are a failure or have let yourself or your family down?

Not at all

Several days

More than half the days

Nearly every day

Trouble concentrating on things, such as reading the newspaper or watching television?

Not at all

Several days

More than half the days

Nearly every day

Moving or speaking so slowly that other people could have noticed? Or so fidgety or restless that you have been moving a lot more than usual?

Not at all

Several days

More than half the days

Nearly every day

Thoughts that you would be better off dead, or thoughts of hurting yourself in some way?

Not at all

Several days

More than half the days

Nearly every day

How difficult have these problems made it for you to perform your work, study, or take care of your household responsibilities, or get along with people?

Not difficult at all

Somewhat difficult

Very difficult

Extremely difficult
